# Resurrection of Oral Arsenic Trioxide for Treating Acute Promyelocytic Leukaemia: A Historical Account From Bedside to Bench to Bedside

**DOI:** 10.3389/fonc.2020.01294

**Published:** 2020-08-04

**Authors:** Cyrus R. Kumana, Raymond Mak, Yok-Lam Kwong, Harinder Gill

**Affiliations:** ^1^Department of Medicine, The University of Hong Kong, Hong Kong, China; ^2^Department of Pharmacy, Queen Mary Hospital, Hong Kong, China

**Keywords:** oral arsenic trioxide, acute promyelocitic leukaemia, history, pharmacokinetics, clinical applications

## Abstract

Various forms of arsenic were used in China and elsewhere for over 5,000 years. Following the initial success of intravenous arsenic trioxide (i.v. As_2_O_3_), we revived an oral formulation of pure As_2_O_3_ in 1998 for the treatment of acute promyelocytic leukemia (APL). We were the first to produce a 1 mg/ml oral-As_2_O_3_ solution and showed that it had comparable bioavailability to i.v. As_2_O_3_. Moreover, we also reported that intracellular arsenic concentrations were considerably higher than the corresponding plasma values. Our oral-As_2_O_3_ was patented internationally and registered in Hong Kong for the treatment of APL. Safety, tolerability and clinical efficacy was confirmed in long-term follow-up studies. We have extended the use of oral-As_2_O_3_ to frontline induction of newly diagnosed APL. With these findings, we are moving toward an era of completely oral and chemotherapy-free management of APL.

## Introduction and Initial Observations

Over many centuries and even millennia, numerous accounts have attested that imbibing arsenicals was a powerful means of poisoning as well as a purported remedy for treating many diseases (1–4). Arsenic first appeared in Western Medicine in the eighteenth century. It was first patented in 1771 by Thomas Wilson for the treatment of malaria and agues. Thomas Fowler from Edinburgh subsequently produced a 1% solution of potassium arsenite, known as “Fowler's solution” (1) From the 1830s to the 1930s, oral arsenic was predominantly used for the management of syphilis, parasitic infestations, chronic skin conditions, and asthma (4). In Hematology, oral arsenic was first reported in the treatment of chronic myeloid leukemia from the 1860s to 1920s in Germany and Boston (1, 4). This practice was phased out following World War II with the development of alkylating chemotherapy and radiotherapy. Oral Fowler's solution, known as “liquor arsenicalis” was produced in Queen Mary Hospital, Hong Kong until the mid-1950s when its use as an anti-leukemic agent was replaced by chemotherapy and radiotherapy (1).

Pure intravenous pure As_2_O_3_ solution was first used in Harbin, China in 1973. Data on the mechanism, pharmacokinetics, and clinical efficacy were extensively published in 1996. Similar treatment results were confirmed around the world (5–7). Moreover, as the Chinese had described using intravenous (i.v.) treatment, the Food and Drug Administration (FDA) in the US agreed to license an American company to produce an i.v. formulation of As_2_O_3_ (Trisenox^®^) for treating APL. Treatment with Trisenox^®^ was inconvenient, cumbersome and prohibitively expensive. Depending on the source, current monthly costs of i.v.-As_2_O_3_-based regimens typically used during induction or re-induction of APL may amount to ~10,000–11,000 U.S. dollars, though more affordable generic formulations are increasingly available (8, 9). Moreover, besides the burdensome quality of life impairments and medication costs of such recurrent i.v. treatment, patients inevitably incurred additional expenses. The latter would be to cover the costs of hospital admissions or day-care attendances, medical and nursing staff, i.v. infusion equipment and fluids, as well as to deal with infusion site-related complications. In addition, the patients would incur necessary travel expenses and loss of earnings due to absence from work.

With memories of Fowler's solution, we revived oral-As_2_O_3_ or the “modern” liquor arsenicalis in 1998 as a means of treating APL patients. This stemmed from two sets of key historical observations. Both of them could be regarded as bedside experiences and inferences that led to laboratory testing and bench-side work, the fruits of which were eventually passed on to patients at the bedside:

1. Meticulously chronicled medical records of Hong Kong CML patients cared for in the 1950s, consistently detailed objective benefits after treatment with Fowler's solution. Accordingly, researchers set out to reinvestigate a possible role for oral-As_2_O_3_ as part of the modern management of APL patients. Treating patients with an oral As_2_O_3_ formulation manufactured in accordance with Good Manufacturing Practice (GMP) could therefore have the potential to confer important benefits with a degree of confidence and safety that was never attained by Fowler's solution.
2. Meanwhile, important bedside observations and correlations arose from advances in molecular genetics, *in-vitro* studies describing As_2_O_3_ induced apoptosis and differentiation of APL cells, and an understanding of chromosomal mutations that accompany aging and disease (10, 11). Notably, APL is almost always associated with the specific chromosomal translocations, *t*(15;17)(q24;q21), and there was mounting evidence that their presence identified patients who respond much more favorably to treatments based on arsenic and all-*trans*-retinoic acid (ATRA) than to conventional treatment (12, 13).

In contrast to using the i.v. route, treatment with a safe and reliable oral-As_2_O_3_ formulation whose production conformed to GMP standards, obviously had the potential to vastly improve quality of life and treatment affordability for APL patients. At the same time, it could also harness the newly realized benefits of avoiding conventional chemotherapy. Another important advantageous distinguishing feature of oral as opposed to i.v. dosing was that it appeared to be less cardiotoxic. Potentially and actually fatal cardiac arrhythmias associated with excessive electrocardiographic QTc interval prolongation were a recognized feature of parenteral treatment (14–16).

## Redeveloping an Oral Formulation of Arsenic Trioxide in Hong Kong

The primary objective of this initiative was to determine whether the systemic bioavailability of an in-house locally developed oral As_2_O_3_ formulation would be comparable to that following commercially available i.v. dosing, when administered to patients with relapsed/refractory APL or acute myeloid leukemia. A secondary objective was to ascertain the extent of arsenic accumulation in the non-cellular and cellular components of blood. However, redeveloping such an oral formulation and determining its systemic bioavailability whilst ensuring acceptability for clinical use in very sick patients posed a number of significant challenges. These challenges and how they were addressed are listed:

1. In the absence of any readily available commercially produced pharmaceutical grade As_2_O_3_ powder, a good quality substitute was eventually sourced from Sigma (U.S.A.).
2. Being a sparingly soluble powder, a suspension was prepared in sterile water and subjected to manipulation of its pH to produce a clear colorless solution with a pH 7.2 that contained 1 mg/ml of As_2_O_3_. Contrary to recommendations in available pharmacopeias, fungicide was not added as the entire preparation process was conducted in a pharmaceutical isolator. Samples subsequently submitted to microbiology and chemical testing yielded no fungi and the solute concentration remained unchanged; its shelf-life exceeded 6 months and very likely extended to more than 1 year.
3. As a means of investigating As_2_O_3_ bioavailability in fairly sick hospitalized patients, the study protocol was necessarily unconventional and did not entail tolerability testing, randomization, a crossover design, or any form of blinding. In all, 9 patients (aged 17–67 years; mean weight of 58 Kg; 6 men and 3 women) were recruited using predefined inclusion/exclusion criteria and asked to refrain from seafood (an arsenic source) in the preceding week. None of them had antecedent renal or liver function test abnormalities. Each patient received a 10 mg i.v. infusion of As_2_O_3_ over 1 h on day 1, followed by a 10 mg oral dose 24 h later. Venous blood samples were drawn from each patient, just before initiating i.v. dosing and at predefined times over the next 48 h. To maximize retrieval of potentially useful data from each sample, aliquots of whole blood and freshly separated plasma were stored and subsequently analyzed in batches using well-established methods.
4. Institutional Ethics Committee approval of the proposed unconventional bioavailability study protocol was achieved after drawing attention to several compelling issues. First, all the patients would have relapsed or refractory disease that was an indication for i.v. As_2_O_3_ treatment and second, they would have to give written informed consent. The committee was also informed that recruiting healthy volunteers to take oral and i.v. arsenic to conduct a formal bioavailability study would prove daunting and nor could it reveal how the oral-As_2_O_3_ would be tolerated by diseased patients.
5. Not surprisingly, publication of the study findings based on such an unconventional protocol was treated with skepticism by many journals. One journal editor however, appreciated the special circumstances constraining administration of arsenic to very sick patients and saw fit to allow publication of the study findings, despite advice to the contrary from some reviewers.

## Oral Arsenic Trioxide Bioavailability Determination and Findings

For each patient, systemic bioavailability following i.v. and oral treatment involved comparison of corresponding area under the curve (AUC) for arsenic concentration vs. time plots attributable to each form of dosing (17). The relevant AUCs were derived using standard computer software incorporating the trapezoidal rule.

**Figure 1:**
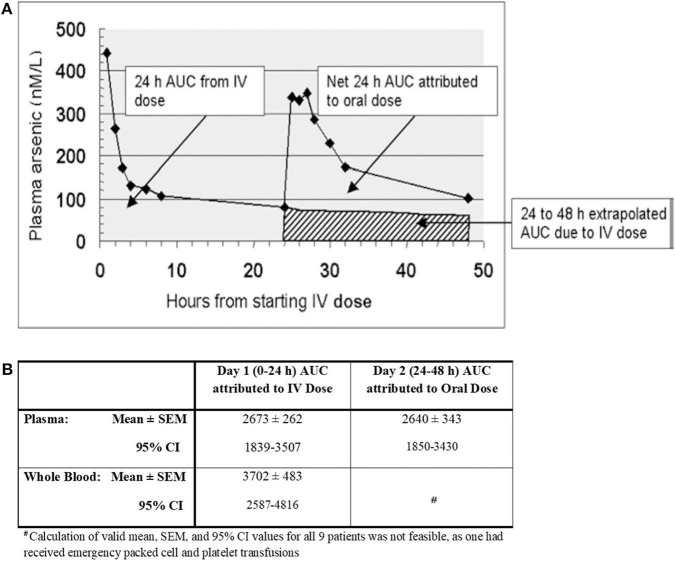
**(A)** Area under the curve (AUC) of arsenic levels attributed to intravenous (i.v.) and oral dosing with arsenic trioxide in a single patient; **(B)** Area Under the Curve (AUC) of Arsenic Concentrations (nanomolar-hours) [adapted from Kumana et al. (17) with permission].

Figure 1 shows plasma arsenic concentrations (in excess of basal levels) prevailing between 0 and 48 h in one representative patient (17). It illustrates how i.v. and oral bioavailability (AUC over the first 24 h following each dose) was inferred, assuming first order terminal elimination of arsenic. The net attributable 0–24 h AUC after oral dosing was taken to be the difference between the gross 24–48 h AUC and the extrapolated 24–48 h AUC attributed to i.v. dosing. In the same way, plasma and whole blood arsenic concentration AUCs were computed for all 9 patients, and the ensuing mean ± standard error of mean (SEM) results and 95% confidence intervals (C.I.) were calculated.

## Clinical Development

These pharmacokinetic studies indicated that, first, the oral formulation and i.v. dosing achieved comparable systemic bioavailability, and secondly, arsenic concentrations in the cellular component of blood were considerably higher than in plasma. Since then, the main finding of this study, namely that orally administered (though differently formulated) As_2_O_3_ attains virtually the same systemic bioavailability as i.v. dosing was confirmed by others (18, 19). The latter researchers also pointed out that compared to i.v. dosing, oral dosing was well-tolerated, more convenient, and equally safe. The observation that arsenic attains higher concentrations in the cellular components of blood than in plasma has also been reported (20). Furthermore, due to arsenic existing as III and V forms, its speciation, pharmacokinetics, and metabolic profiling is complex and confusing (10, 19–22). Yet, based on the latter available pharmacokinetic findings, there are reasonable grounds for accepting that the terminal phase of arsenic elimination from plasma approximates to a first order process, and supports extrapolation of 24–48 h AUCs attributable to i.v. dosing. As indicated by the CIs detailed in Figure 1B, there was substantial inter-patient variation in both plasma and whole blood AUCs. Though not tabulated for individual patients, up to ~5 and 10-fold variations, respectively, were encountered between individuals, whilst inter-day variation was much less marked. Interestingly, since considerable inter-patient plasma level/AUC variations have also been encountered after i.v. dosing (21, 22), differences in arsenic absorption capacity from the gut is unlikely to be the main explanation. Thus, conceivable reasons for such variations include saturable tissue binding of As_2_O_3_, dietary indiscretions by individual patients, and inherent disease state/physiological differences. It is nevertheless evident that repeated courses of oral As_2_O_3_ with these types of therapeutic doses are safe (18, 19, 23). The optimal dose of oral-As_2_O_3_ we suggest is 10 mg (0.15-0.2 mg/kg) per day in adult patients (≥50 kg) with normal renal function. In adults weighing less than 50 kg and in pediatric patients, oral-As_2_O_3_ solutions at 0.15 mg/kg are advised. With plasma and intracellular arsenic level monitoring, we were also able to administer oral-As_2_O_3_ safely at a lower doses for patients with end-stage renal failure or patients dialysis (24, 25).

Interestingly, standard i.v.-As_2_O_3_ dosing regimens have been repeatedly incriminated as a cause of cardiac arrhythmias and sudden death, possibly associated with electrocardiographic QTc interval prolongation induced by arsenic (14–16). The latter phenomena have been linked to excessive levels of plasma arsenic. Oral dosing seems to mitigate this cardiac risk, possibly because arsenic's entry into the circulation from the gut is much more gradual and the peak concentrations attained are consequently much lower (26). Based on having demonstrated this particular likely pharmacokinetic advantage of the aforementioned oral-As_2_O_3_ formulation, in 2009 its inventors were granted a U.S. patent for treating APL patients. Thereafter, patents for this formulation were also granted by the European Union, China, and Japan. Nevertheless, it should be noted that there has been a single case report of such cardiac arrhythmias occurring transiently after oral therapy of a patient with known chemotherapy-induced dilated cardiomylopathy (27).

A plethora of well-conducted phase 2 studies followed. Our group demonstrated excellent long-term outcomes in APL patients treated with oral-As_2_O_3_-based regimens (28–31). In a 15-year prospective follow-up study in 73 patients with relapsed APL, idarubicin (6 mg/m^2^/day for 5 days) plus oral-As_2_O_3_ (10 mg/day), all-trans-retinoic acid (45 mg/m^2^/day) and ascorbic acid (1 g/day) (AAA) for 42 days resulted in a 100% molecular remission rate (28). Ascorbic acid was used in the AAA regimen due to its synergism with As_2_O_3_ which has been shown *in-vitro* and clinically (30, 32, 33). Following second complete remission (CR2), 2 monthly cycles of idarubicin (6 mg/m^2^/day for 3 days) plus AAA for 7 days followed by 12 cycles of AAA maintenance (given for 2 weeks every 2 months for 2 years) resulted in 5-year and 10-year overall survival (OS) of 79.5 and 67.3%, respectively (28). Importantly, this was achieved without hematopoietic stem cell transplantation (HSCT) in CR2. This shows that prolonged AAA maintenance is an effective post-remission strategy following CR2, obviating the need for HSCT, a procedure still considered a standard for managing such patients in many places around the world. We then moved AAA forward as post-remission maintenance following CR1, which resulted in a 5-year leukemia-free survival (LFS) and OS of 90% and 97%, respectively (29–31). Most recently, we incorporated AAA (given for 42 days) into frontline induction for newly diagnosed APL with daunorubicin (50 mg/m^2^/day for 3 days) followed by 2 cycles of consolidation with daunorubicin (50 mg/m^2^/day for 2 days) and cytarabine (100 mg/m^2^/day for 5 days) and 2 years of AAA maintenance. Both LFS and OS were 100% at 5 years (29). In patients aged 70 or above or those with medical co-morbidities, chemotherapy was omitted and patients were treated with an entirely oral regimen comprising 42 days of AAA and no relapses have been observed so far (29). With AAA-based regimens, outcome for both newly diagnosed and relapsed APL were independent of the conventional risk scores. With LFS plateauing 2 years after completion of maintenance both in CR1 or CR2, long-term molecular monitoring is not necessary 2 years beyond completion of AAA maintenance following CR1 or CR2 (28, 29). Even in conventional high-risk patients, our strategy of incorporating oral-As_2_O_3_ to frontline induction achieved excellent long-term outcomes similar to those achieved in low-risk patients. It remains to be seen whether maintenance is necessary in low-risk patients treated with frontline oral-As_2_O_3_-based induction. We are currently testing frontline induction with AAA in APL (ClinicalTrials.gov Identifier: NCT03624270) in a risk-adapted manner incorporating a chemotherapy-free approach.

In our oral-As_2_O_3_ studies, both short-term and long-term cardiac safety was confirmed. QTc prolongation, ventricular arrhythmias and cardiac failure were not observed. QTc prolongation occurred in 16% of patients given i.v. As_2_O_3_ which was significantly higher than that observed with our regimen (12, 34, 35). We have demonstrated lower peak plasma arsenic levels with oral-As_2_O_3_ dosing that probably accounted for the cardiac safety. Drug-induced transaminitis (Grade 1–2: 31%; Grade 3–4: 26%) were all reversible with transient dose reductions or interupptions (29). Upon normalization of liver enzymes, all patients were able to tolerate oral-As_2_O_3_ at 10 mg/day without recurrence of transaminitis. Our regimen of AAA showed similar or lower rates of hepatoxicity than with i.v. As_2_O_3_-ATRA treatment, the latter being associated with transaminitis that occurred in 44–71% of patients (12, 34, 35). Acyclovir prophylaxis is used universally in all patients on oral-As_2_O_3_ due to the risk of herpes zoster that was demonstrated in our earlier studies (36). Differentiation syndrome (DS) occurred in 26 and 12% in patients with newly diagnosed APL and APL in first relapse (R1), respectively, whilst no induction deaths were observed. With early recognition, cytoreduction and the use of dexemathasone, interruption of oral-As_2_O_3_ or ATRA was not necessary in our studies. Other common side-effects of the AAA regimen included headache (Grade 1–2: 32%) and upper gastrointestinal upset (Grade 1–2: 11%) most of which were either self-limiting or controlled with simple analgesics and antacids.

In our studies involving relapsed APL patients receiving oral-As_2_O_3_, as with i.v.-As_2_O_3_ treatment – there was a high risk of central nervous system disease in those with severe relapses (28, 37). We also showed that after an oral administration, meaningful cerebrospinal fluid (CSF) levels of arsenic were achieved implying its benefit in the prophylaxis or treatment of central nervous system (CNS) disease (38, 39). CSF and plasma arsenic levels were linearly correlated with CSF arsenic levels at about 18% of the levels in plasma levels (38). Frontline use in newly diagnosed APL may ameliorate the risk. Since 2013, none of our patients treated with frontline oral-As_2_O_3_ had evidence of CNS relapse (29). There is limited data on CSF arsenic levels in patients treated with i.v. As_2_O_3_. The concurrent use of intravenous mannitol may significantly increase CSF arsenic levels comparable to those in blood, thus providing the prospect of managing CNS APL effectively (40).

## Other Oral Arsenic Preparations

Realgar-Indigo naturalis formula (RIF) was developed in the 1980s entirely based on traditional Chinese Medicine (TCM) concepts and comprises realgar, *Indigo naturalis, Salvia miltiiorrhiza* and *Radix psudostellariae* (40) and was launched in Mainland China in 2009. Tetraarsenic tetrasulphide (As_4_S_4_), indirubin and tanshinone IIA are the active ingredients of RIF, which show in-*vitro* synergism (40, 41). RIF showed comparable efficacy to i.v. As_2_O_3_ (42, 43), together with improved quality of life and reduced costs. Hepatotoxicity is frequently reported and occurs in about 58–65% (Grade 1–2: 49–55%; Grade 3–4: 9–10%) of patients treated with RIF plus ATRA (42, 43). Another common toxicity of RIF is diarrhoea which occurs in about 15% of patients. Prolonged QTc intervals are uncommon with RIF at dosages of 60 mg/kg/day but occurs in about 24% of patients given a high dose of 7.5 g/day (44). Clinically significant arrhythmias are nevertheless rare. Groups in Australia (ANZCTR registration number: ACTRN12616001022459 and the United States (ClinicalTrials.gov Identifier: NCT03048344) have developed novel formulations of oral As_2_O_3_. For instance, ORH-2014 was recently shown to be safe in patients with leukaemia and had comparable bioavailability to i.v. As_2_O_3_ (19).

## Conclusion

**Figure 2:**
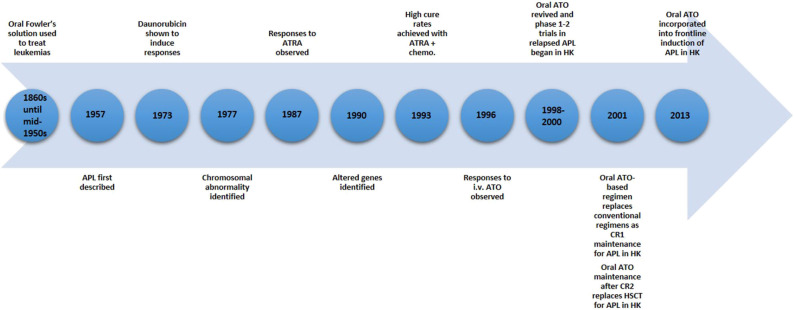
Timeline of advances in acute promyelocytic leukaemia (APL) treatment and the development of oral arsenic trioxide (ATO) in Hong Kong. ATRA, all-*trans*-retinoic acid; chemo., chemotherapy; HK, Hong Kong; i.v., intravenous; CR1, first completeremission; CR2, second complete remission; HSCT, hematopoietic stem cell transplantation.

The history of APL and development of arsenic trioxide has formed a paradigm for targeted therapy in cancer (Figure 2). There is every reason to believe that the form of oral treatment described above can offer therapeutic benefits equivalent to dosing with i.v. As_2_O_3_, with the added advantages of far superior convenience (enabling injection free home treatment with outpatient supervision), greater affordability, and a lower risk of cardiac incidents. Moreover, though reverting to oral treatment can be considered as only a small incremental step in the overall context of treating haematogical malignancies such as APL, for individual patients it can nevertheless be regarded as an important and life-changing advance. A commercially available oral-As_2_O_3_ in the not-too-distant future will pave the way for this inexpensive and convenient form of As_2_O_3_ to be available worldwide.

## Author Contributions

CK, Y-LK, and HG: conception, manuscript writing, and final approval of manuscript. RM: manuscript writing and final approval of manuscript. All authors: contributed to the article and approved the submitted version.

## Conflict of Interest

The University of Hong Kong currently holds two United States (US) patents (7,521,071 B2 and 8,906,422 B2), one Japanese patent (4786341) and one European patent (EP 1562616 B1) for the use of oral-As_2_O_3_ in the treatment of leukemias and lymphomas. HG, CK, and Y-LK are employed by or associated with the University of Hong Kong. The remaining author declares that the research was conducted in the absence of any commercial or financial relationships that could be construed as a potential conflict of interest.
